# Bradycardia, Renal Failure, Atrioventricular Nodal Block, Shock, and Hyperkalemia (BRASH) Syndrome-Induced Atrial Fibrillation: A Case Report

**DOI:** 10.7759/cureus.59057

**Published:** 2024-04-26

**Authors:** Allan E Santos Argueta, Junaid Ali, Parthiv Amin

**Affiliations:** 1 Internal Medicine, Saint Peter’s University Hospital, New Brunswick, USA; 2 Interventional Cardiology, Saint Peter’s University Hospital, New Brunswick, USA

**Keywords:** hyperkalemia-induced ekg changes, cardiac electrophysiology, sever hyperkalemia, new onset afib, brash syndrome

## Abstract

BRASH syndrome is a syndrome that comprises bradycardia, renal failure, atrioventricular nodal block, shock, and hyperkalemia. This syndrome is usually associated with a junctional rhythm. Early recognition of this clinical entity is crucial for appropriate management. In this case report, we describe a 70-year-old female who presented with BRASH syndrome-induced atrial fibrillation with a slow ventricular response.

## Introduction

BRASH syndrome consists of bradycardia, renal failure, atrioventricular nodal block, shock, and hyperkalemia. It describes a cycle in which atrioventricular (AV) node-blocking agents cause bradycardia and hyperkalemia, leading to decreased cardiac output, manifesting in shock and kidney injury. This continuum cycle worsens renal function and hyperkalemia, which can eventually lead to multi-organ involvement [1]. Electrocardiogram (EKG) in BRASH syndrome is usually associated with junctional rhythm, bradycardia, or AV block; however, rare cases have been reported with EKG showing tachyarrhythmias [2]. In this case report, we describe a patient presenting with BRASH syndrome with an EKG showing atrial fibrillation (AFib) with a slow ventricular response.

## Case presentation

**Figure 1:**
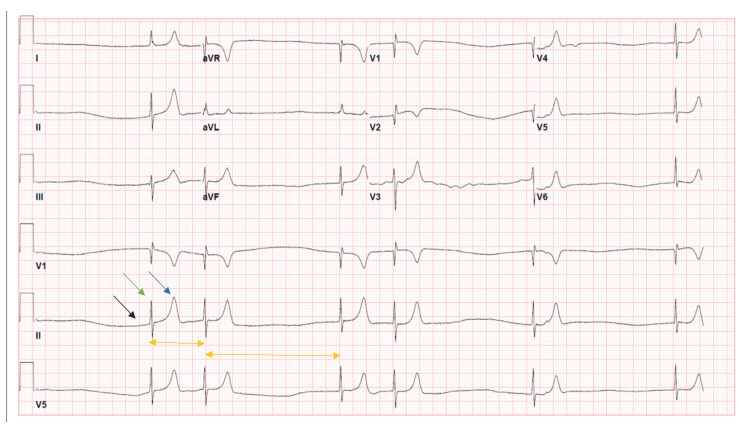
EKG on admission *Black arrow*: absence of the P wave; *green arrow:* normal QRS complex; *blue arrow:* peaked T wave; *yellow arrows*: irregular QRS intervals aVR: augmented vector right; aVL: augmented vector left; aVF: augmented vector foot

A 70-year-old female with hypertension and type 2 diabetes mellitus, who is taking losartan 50 mg, metoprolol succinate 50 mg, amlodipine 5 mg, rosuvastatin 10 mg, and insulin therapy, presented to the hospital with fatigue, lightheadedness, and palpitations for one day. In the emergency room, she was found hypotensive with a blood pressure of 72/35 mmHg and an abnormally erratic heart rate of 40 beats per minute with no signs of volume overload on the physical exam. Atrial fibrillation with a slow ventricular response was noted in the EKG with associated peak T waves (Figure 1). The laboratory values throughout the admission are shown in Table 1.

**Table 1:** Laboratory results BUN: blood urea nitrogen; eGFR: estimated glomerular filtration rate

	Baseline	On Admission	On Discharge	Reference Range
BUN	14	29	15	9-28 mg/dL
Creatinine	0.66	1.5	0.7	0.52-1.04 mg/dL
eGFR	87	33	81	>60
Glucose	207	500	151	82-115 mg/dL
Sodium	136	133	139	136-145 mmol/L
Potassium	4.2	7.8	4.4	3.5-5.1 mmol/L
Chloride	102	99	106	99-112 mmol/L
Bicarbonate	25	25	27	21-33 mmol/L
Anion gap	9	9	8	4-12

**Figure 2:**
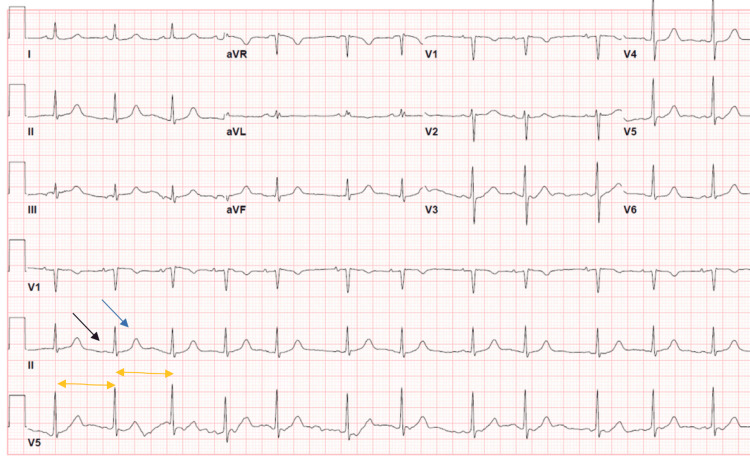
EKG on discharge *Black arrow:* presence of the P wave; *blue arrow:* normal T wave; *yellow arrows:* regular QRS interval aVR: augmented vector right; aVL: augmented vector left; aVF: augmented vector foot

Initial management in the emergency room consisted of a trial of three doses of 1 mg of atropine as per advanced cardiac life support (ACLS) protocol and IV fluid boluses with minimal improvement. She received calcium gluconate, and posteriorly, she was managed in the ICU with insulin infusion, albuterol, sodium bicarbonate, and sodium polystyrene. Cardiology was consulted, given the CHA_2_DS_2_-VASc score of 4 points, and the recommendation against anticoagulation was made, given that new-onset AFib is likely secondary to severe hyperkalemia and acute illness. Her creatinine level improved to baseline, achieving hemodynamic stability and normalizing potassium levels. A repeat EKG within 48 hours since her admission showed normal sinus rhythm (Figure 2). She was safely discharged and advised to follow up with Cardiology for further monitoring after her spontaneous conversion to sinus rhythm.

## Discussion

The toxicity of potassium is linked to its effect on the myocardium by reducing conduction velocity and accelerating the repolarization phase, which is initially noted as peaking T waves and progressing to loss of P waves with widening of the QRS complexes, and in more severe hyperkalemia as profound bradycardia, AV block, or sinus arrest [3].

BRASH syndrome was first described in the late 2010s in patients presenting with nonspecific symptoms due to hypoperfusion, such as generalized fatigue, headache, dizziness, and syncope. It is often precipitated by medications affecting the conduction system of the heart, specifically the AV node, such as beta blockers and non-dihydropyridine calcium channel blockers; however, cases of amiodarone and octreotide have also been reported [4]. The effect of both hyperkalemia and AV node blocking synergically on the heart rate leads to a progressive decline in cardiac output, manifesting in shock and acute renal failure [2]. This leads to decreased renal clearance of AV-blocking agents and worsening hyperkalemia, repeating the cycle [5].

Given the nonspecific symptoms and signs, a high level of clinical suspicion is warranted for diagnosis. The most commonly reported electrographic findings are junctional rhythm and bradycardia, with or without the characteristic EKG changes of hyperkalemia. Laboratory findings include a significantly elevated creatinine and moderate to severe hyperkalemia with or without other electrolyte abnormalities [6,7].

Treatment aims to stabilize the cardiac membrane with calcium gluconate and hemodynamic support with IV fluids and vasopressors if required. Underlying hyperkalemia needs to be managed with agents associated with reducing total body potassium [5,8]. Advanced therapies are reserved for patients refractory to treatment, requiring dialysis and/or temporary transvenous pacing whenever initial management fails [1,9].

As per the 2023 ACC/AHA/ACCP/HRS guidelines for AFib, the recurrence of AFib is significant in acute medical illnesses requiring outpatient follow-up for surveillance. In these circumstances, the benefits of anticoagulation for stroke prevention are not well-defined [10].

## Conclusions

BRASH syndrome requires early recognition to enable prompt correction of its underlying cycle and improve the prognosis. This case highlighted an atypical EKG finding in a patient diagnosed with BRASH syndrome who was not started on anticoagulation, given the reversible underlying condition in an acute illness setting. Further studies are required to determine the long-term outcomes of recurrence and thromboembolic events in patients with paroxysmal AFib induced by BRASH syndrome.
